# Growth, body composition and bone mineral density among pubertal male athletes: intra-individual 12-month changes and comparisons between soccer players and swimmers

**DOI:** 10.1186/s12887-022-03321-2

**Published:** 2022-05-13

**Authors:** Daniela C. Costa, João Valente-dos-Santos, Paulo Sousa-e-Silva, Diogo V. Martinho, João P. Duarte, Oscar M. Tavares, Joaquim M. Castanheira, Tomás G. Oliveira, Sandra Abreu, Neiva Leite, Ricardo R. Agostinete, Rômulo A. Fernandes, Daniel Courteix, Manuel J. Coelho-e-Silva

**Affiliations:** 1 University of Coimbra, FCDEF, Coimbra, Portugal; 2 CIDAF (uid/dtp/04213/2020), University of Coimbra, Coimbra, Portugal; 3 Lusophone University of Humanities and Technologies, CIDEFES, Lisbon, Portugal; 4 Polytechnic Institute of Coimbra, Coimbra Health School, Coimbra, Portugal; 5 University of Porto, CIAFEL, Porto, Portugal; 6 Department of Physical Education, Federal University of Parana, Curitiba, PR Brazil; 7 Department of Physical Education, Laboratory of InVestigation in Exercise (LIVE), Sao Paulo State University (UNESP), Presidente Prudente, Brazil; 8 Laboratory AME2P, University of Clermont Auvergne, Clermont-Ferrand, France; 9 Estadio Universitario, Pavilhao III, 3040-248 Coimbra, Portugal

**Keywords:** DXA, Mechanical loading, Bone health, Body composition, Youth sport

## Abstract

**Background:**

Puberty is a period of intense changes in human body and, additionally, participation in sports is viewed as prominent form of physical activity among male adolescent athletes. The current study was aimed to examine the intra-individual changes in body composition and bone tissue during years of maximal growth and the effect of 12-month participation in sports contrasting in mechanical impact.

**Methods:**

The sample included 40 male adolescent athletes (soccer: *n* = 20; swimming: *n* = 20) aged 12.57 ± 0.37 years who were followed for 12 months. Stature and body mass were measured, bone mineral content (BMC), areal bone mineral density (aBMD), lean soft and fat tissues assessed using DXA. Food intake was estimated using a questionnaires and training sessions individually monitored. Repeated measures ANOVA tested the differences between sports and 12-month intra-individual variation (time moments: TM1, TM2). The analyses on aBMD for total body and total body less head were repeated controlling for variation in stature at baseline.

**Results:**

Soccer players completed 63 ± 31 sessions (95 ± 47 h). Respective values for swimmers were 248 ± 28 sessions and 390 ± 56 h. In general, the analysis of aBMD as dependent variable evidenced significant effect of sport-associated variation (*F* = 5.254, *p* < 0.01; η^2^ = 0.35) and 12-month increments, particularly at lower limbs (*F* = 97.238, *p* < 0.01; η^2^ = 0.85). Respective mean values for aBMD were SCC_TM1_ = 0.885 g.cm^−2^, SWM_TM1_ = 0.847 g.cm^−2^, SCC_TM2_ = 0.939 g.cm^−2^, SWM_TM2_ = 0.880. Regarding the lean soft tissue, the magnitude of effects was very large for intra-individual variation (*F* = 223.043, *p* < 0.01; η^2^ = 0.92) and moderate between sports (*F* = 7.850, *p* < 0.01; η^2^ = 0.41): SCC_TM1_ = 30.6 kg, SWM_TM1_ = 34.9 kg, SCC_TM2_ = 35.8 kg, SWM_TM2_ = 40.5 kg). Finally, d-cohen values reporting percentage of intra-individual changes in aBMD between soccer players ad swimmers were large for the trochanter (d = 1.2; annual increments: SCC = 8.1%, SWM = 3.6%).

**Conclusion:**

Puberty appeared as a period of significant intra-individual changes in lean soft tissue and bone mineral density. With increasing accumulated training experience, mean difference between sports contrasting in mechanical impact tended to me more pronounced in particular at the lower limbs.

**Supplementary Information:**

The online version contains supplementary material available at 10.1186/s12887-022-03321-2.

## Background

It is estimated that 85–90% of the adult bone mass is gained during the first two decades of life [1, 2]. During years of maximal growth velocity, physical activity is directly associated to gains in peak bone mass [3] and afterwards, during adulthood, weight-bearing activities are supposed to improve bone strength and maintain bone mass [4]. The skeleton is sensitive to transverse and torsional forces directly applied on bones in addition to mechanical stimulus derived from muscle contractions. The preceding are essential characteristics of sports which is probably the main form of physical activity in young people involving jumps, sprints, changes of directions and many physical contact [5, 6]. By inference, during circumpubertal years, sport participation is hypothesized to assume an important influence on bone health. The type of sports may be a source of variation in developmental changes of the bone tissue.

Dual energy x-ray absorptiometry (DXA) is probably the most used instrument to obtain indicators of bone tissue and the technology is also being used in the assessment of total and regional body composition [7]. In addition, DXA allows information about the proximal femur which is considered a region of interest to prevent osteoporotic fractures [8, 9]. Recent research on adolescent athletes considered the above cited region [10, 11]. Nevertheless the ambivalent characteristics of DXA, studies did not systematically consider the interrelationship among bone tissue and variation in body composition. During circumpubertal years, variation in body composition is essentially attributable to growth, maturation [12] and, in the case of youth athletes, type, frequency and duration of training sessions are also plausible longitudinal predictors.

In Portugal, as in many other countries [13], soccer and swimming consistently merge among the most popular sports. Swimming is classified as hypogravity and requests propulsive forces mostly produced by the upper body [14]. In parallel, soccer is characterized by high-intensity intermittent efforts demanding repeated actions of the lower limbs: jumps, sprints, changes of direction, tackles [15, 16]. Recent research [11] evidenced weight bearing sports such as soccer, basketball or volleyball had a positive osteogenic effect given by increasing levels of bone mineral content (BMC) and areal bone mineral density (aBMD). Meantime, another study concluded that participation in soccer induced significantly greater improvements in BMC and bone stiffness over 12 months compared to cycling and swimming [17]. The preceding was derived from a 12-month study conducted in 116 adolescent males engaged in soccer, swimming and cycling who were compared with an active control group. Another study reporting young athletes from non-weight bearing sports such as swimming and cycling did not present any substantial osteogenic effect [6].

Research investigating the independent and combined effects of sports and 12-month training on bone development in adolescent males is still scarce, particularly considering adjustment for stature and including parameters of body composition. Of relevance, soccer and swimming correspond to distinct training routines regarding the number of training sessions and weekly volume and this supposedly has impact on body composition. Unfortunately, the literature did not systematically collect data about training experience. Taking into account the previous, this study was aimed to examine the effect of sport over a 12-month season among male adolescent soccer players and swimmers on body size, body composition and indicators of bone health. It was hypothesized that adolescent athletes from sports contrasting in mechanical impact differ in terms of inter- and intra-individual bone aBMD with intra-individual gains more pronounced at the proximal femur.

## Methods

### Study design and procedures

The current research was approved by the Ethics Committee for Sports Sciences of the University of Coimbra (CE/FCDEF-UC/00182016) following the Declaration of Helsinki for human studies [18]. Participants were male adolescent athletes recruited from Portuguese clubs. Signed informed consents were obtained from parents or legal guardians. Participants were informed about the objectives, procedures, benefits, risks and also that they could withdraw from the study at any time. All data was collected within a 12-month period (baseline or TM1; 12-month follow-up or TM2) under standardized conditions at the same laboratory by the same observers and instruments.

### Participants

This project contacted 12 clubs that allow participation in soccer and swimming for 262 male adolescents. Eighty-one male adolescents visited the laboratory. Exclusion criteria were: [i] registered in competitive sports at respective Portuguese federation for less than two complete seasons at baseline: 10 soccer and 6 swimmers were excluded; [ii] failed to be classified as pre-peak height velocity (PHV) based on estimated PHV, i.e., positive predicted maturity-offset values at baseline excluded 9 soccer players and 8 swimmers; [iii] presence of fractures, chronic diseases, eating disorders or medication that could have affected bone metabolism: any exclusion, [iv] eight participants failed the second visit for repeated measurements: 3 soccer players and 5 swimmers. After considering the inclusion criteria, the sample was composed of 40 participants (soccer players: *n* = 20; swimmers: *n* = 20). By using G*Power software (v3.1.9.2, University of Kiel, Germany), two groups (soccer *vs.* swimming), measured twice (baseline *vs.* after 12 months) and including a covariate correspond to a power sample equal to 87%.

### Chronological age and training experience

Chronological age (CA) was calculated to the nearest 0.1 year; Training experience was obtained at the TM1 and expressed in years. Information regarding the number of training sessions and accumulated minutes of training were individually collected by contacting coaches and assistant coaches on a regular basis.

### Anthropometry

A single observer measured stature, body mass and sitting height following standardized protocols [19]. Stature and sitting height were measured to the nearest 0.1 cm using a portable stadiometer (Harpenden model 98.603, Holtain LTD, Crosswell, UK) and sitting height table (Harpenden model 98.607, Holtain LTD, Crosswell, UK). Leg length was calculated as the difference of stature and sitting height. Body mass was measured to the nearest 0.1 kg using a portable balance (SECA model 770, Hanover, MD, USA). Body mass index was calculated.

### Biological maturation

A non-invasive indicator of biological maturation was used to confirm that all participants were pre-PHV at the baseline. Maturity offset was obtained from an algorithm requiring CA, body mass, stature, sitting height and estimated leg length. It refers to the distance (in years) to age peak height [20] as presented in Eq. 1.

1 $$\mathrm{Maturity}\ \mathrm{offset}\ \left(\mathrm{years}\right):-9.236+\left(0.0002708\ast \left(\mathrm{leg}\ \mathrm{length}\ast \mathrm{sitting}\ \mathrm{height}\right)\right)+\left(-0.001663\ast \left(\mathrm{CA}\ast \mathrm{leg}\ \mathrm{length}\right)\right)+\left(0.007216\ast \left(\mathrm{CA}\ast \mathrm{sitting}\ \mathrm{height}\right)\right)+\left(0.02292\ast \left(\left[\mathrm{body}\ \mathrm{mass}/\mathrm{stature}\right]\ast 100\right)\right)$$

### Food intake

A semi-quantitative food frequency questionnaire (FFQ) was used to estimate dietary intake validated for Portuguese population [21, 22]. The FFQ included food groups, beverage categories and frequency intake with nine qualitative options (from “never or less than once a month” to “6 or more times per day”). Calories, macronutrients intake, cholesterol, fiber and calcium were estimated using the software Food Processor SQL (ESHA Research Inc., Salem, OR, USA) and, subsequently, were retained for the analysis.

### Dual energy x-ray absorptiometry

Body composition was examined as the sum of fat tissue, LST, BMC using DXA (Hologic QDR-4500 scanner, version 9.10, Hologic Inc., Bedford, Massachusetts, USA). A single certified technician extracted the data following the guidelines published by the manufacturer [23]. Participants were positioned on the table in supine position with the body aligned along with the central axis. This scan permits the calculations of BMC, bone area, and aBMD in addition to fat tissue and LST. The data collection was repeated for the whole body, trunk, upper limbs and lower limbs. The analysis of the proximal femur required a second scan in the non-dominant leg in order to assess aBMD for the femoral neck, ward triangle, trochanter.

### Analysis

Means and standard deviations by sport separately for the baseline and 12-month follow-up were calculated for body size, body composition in addition to aBMD. The subsamples presented identical characteristics for CA at baseline (soccer: 12.4 ± 0.3 years; swimmers: 12.7 ± 0.4 years) and also for maturity-offset (soccer: -1.6 ± 0.5 years; swimmers: -1.2 ± 0.6 years). Consequently, repeated measures analysis of variance (ANOVA) was used to test the effect of sports, intra-individual 12-month changes and interaction term sport*12-month. Meantime, swimmers were slightly taller than soccer players and the repeated measures ANCOVA (controlling for stature at baseline) was performed for two aBMD outputs: total body and subhead, i.e., total body less head. The preceding was recommended for children and adolescents [24]. The effect size for each factor (12-month participation, type of sports, interaction) was given by eta squared that was interpreted as follows [25]: η^2^˂0.1 (trivial), 0.1 ≤ η^2^˂0.3 (small), 0.3 ≤ η^2^˂0.5 (moderate), 0.5 ≤ η^2^˂0.7 (large), 0.7 ≤ η^2^˂0.9 (very large), 0.9 ≤ η^2^ (nearly perfect). Intra-individual differences were individually calculated and expressed as percentage of baseline values and based on means and standard deviation of intra-individual changes for soccer players and swimmers Cohen d-values were calculated [26] and interpreted as follows [25]: d˂0.2 (trivial), 0.2 ≤ d < 0.6 (small), 0.6 ≤ d < 1.2 (moderate), 1.2 ≤ d < 2.0 (large), 2.0 ≤ d < 4.0 (very large) and d ≥ 4.0 (nearly perfect). Finally, descriptive statistics were calculated by sport for body mass index and indicators derived from the food questionnaire in addition to comparisons between groups using student t-test. Statistical significance was set at 5%. All analyses were performed using SPSS version 20.0 (SPSS Inc., IBM Company, N.Y., USA) and Graphpad Prism (version 5.00 for Windows, GraphPad Software, San Diego California USA, www.graphpad.com).

## Results

Table 1 summarizes descriptive statistics separately for the two groups. Soccer players and swimmers significantly differed on lean soft tissue (LST) at all sites with swimmers always presenting higher mean values: total body (*F* = 7.850, *p* < 0.01, η2 = 0.41), trunk (*F* = 8.186, *p* < 0.01, η2 = 0.42), upper limbs (*F* = 11.598, *p* < 0.01, η2 = 0.48) and lower limbs (*F* = 4.371, *p* < 0.05, η2 = 0.32). During the follow-up period, on average, soccer players completed 63 ± 31 training sessions corresponding to 5696 ± 2808 min. Respective values for swimmers were 248 ± 28 training sessions and 23,378 ± 3368 min. Significant differences were also noted between soccer players and swimmers for aBMD on total body (*F* = 5.545, *p* < 0.05, η2 = 0.36) and lower limbs (*F* = 9.146, *p* < 0.01, η2 = 0.44). In the preceding sites, aBMD means of soccer players exceeded the means of the swimmers. Regarding the parameters of the proximal femur, the effect of sports was always significant: femoral neck (*F* = 5.052, *p* < 0.05, η2 = 0.34), ward triangle (*F* = 8.129, *p* < 0.01, η2 = 0.42) and trochanter (*F* = 7.718, *p* < 0.01, η2 = 0.41). Again, soccer players were characterized by higher mean values in aBMD compared to swimmers.

**Table 1:** Descriptive statistics (mean ± standard deviation) by sports and time moment in addition to estimated means for aBMD of the total and subhead (standard error) controlling for variation in stature among male adolescent athletes (soccer players, *n* = 20; swimmers, *n* = 20)

Dependent variable	Groups			
	Soccer (*n* = 20)		Swimming (*n* = 20)	
	Baseline	12-month	Baseline	12-month
	mean ± standard deviation			
Training minutes		5696 ± 2808		23,378 ± 3368
Training sessions		63 ± 31		248 ± 28
Stature, cm	149.8 ± 5.9	157.8 ± 7.1	154.8 ± 7.5	161.2 ± 8.2
Body mass, kg	41.6 ± 7.2	46.8 ± 8.6	44.1 ± 7.4	49.0 ± 7.1
Fat tissue, %	18.0 ± 7.6	17.1 ± 7.9	14.0 ± 8.5	11.6 ± 7.8
LST: total body, kg	30.6 ± 3.8	35.8 ± 5.6	34.9 ± 5.2	40.5 ± 6.2
LST: trunk, kg	13.75 ± 1.76	16.20 ± 2.72	15.83 ± 2.63	18.68 ± 3.09
LST: upper limbs, kg	2.91 ± 0.43	3.47 ± 0.59	3.53 ± 0.63	4.16 ± 0.80
LST: lower limbs, kg	10.81 ± 1.58	12.84 ± 2.25	12.13 ± 1.93	14.16 ± 2.37
aBMD: total body, g.cm^−2^	0.993 ± 0.041	1.027 ± 0.055	0.956 ± 0.065	0.976 ± 0.074
	1.001 (0.012)^a^	1.037 (0.014)^a^	0.948 (0.012)^a^	0.966 (0.014)^a^
aBMD: subhead, g.cm^−2^	0.885 ± 0.050	0.939 ± 0.069	0.847 ± 0.073	0.880 ± 0.077
	0.896 (0.013)^a^	0.952 (0.015)^a^	0.636 (0.013)^a^	0.867 (0.015)^a^
aBMD: trunk, g.cm^−2^	0.806 ± 0.041	0.857 ± 0.061	0.799 ± 0.072	0.843 ± 0.077
aBMD: upper limbs, g.cm^−2^	0.684 ± 0.110	0.698 ± 0.053	0.667 ± 0.046	0.688 ± 0.061
aBMD: lower limbs, g.cm^−2^	1.056 ± 0.077	1.122 ± 0.094	0.987 ± 0.091	1.016 ± 0.106
aBMD: PF-neck, g.cm^−2^	0.950 ± 0.084	1.013 ± 0.104	0.895 ± 0.111	0.923 ± 0.115
aBMD: PF-ward, g.cm^−2^	0.941 ± 0.105	1.003 ± 0.128	0.849 ± 0.139	0.866 ± 0.139
aBMD: PF-trochanter, g.cm^−2^	0.822 ± 0.078	0.889 ± 0.099	0.762 ± 0.099	0.788 ± 0.097

*LST* (Lean soft tissue), *aBMD* (Areal bone mineral density), *PF* (Proximal femur)

^a^(Repeated measures ANCOVA controlling for stature at baseline, centered stature = 152.3 cm, estimated means and associated standard error)

**Fig. 1:**
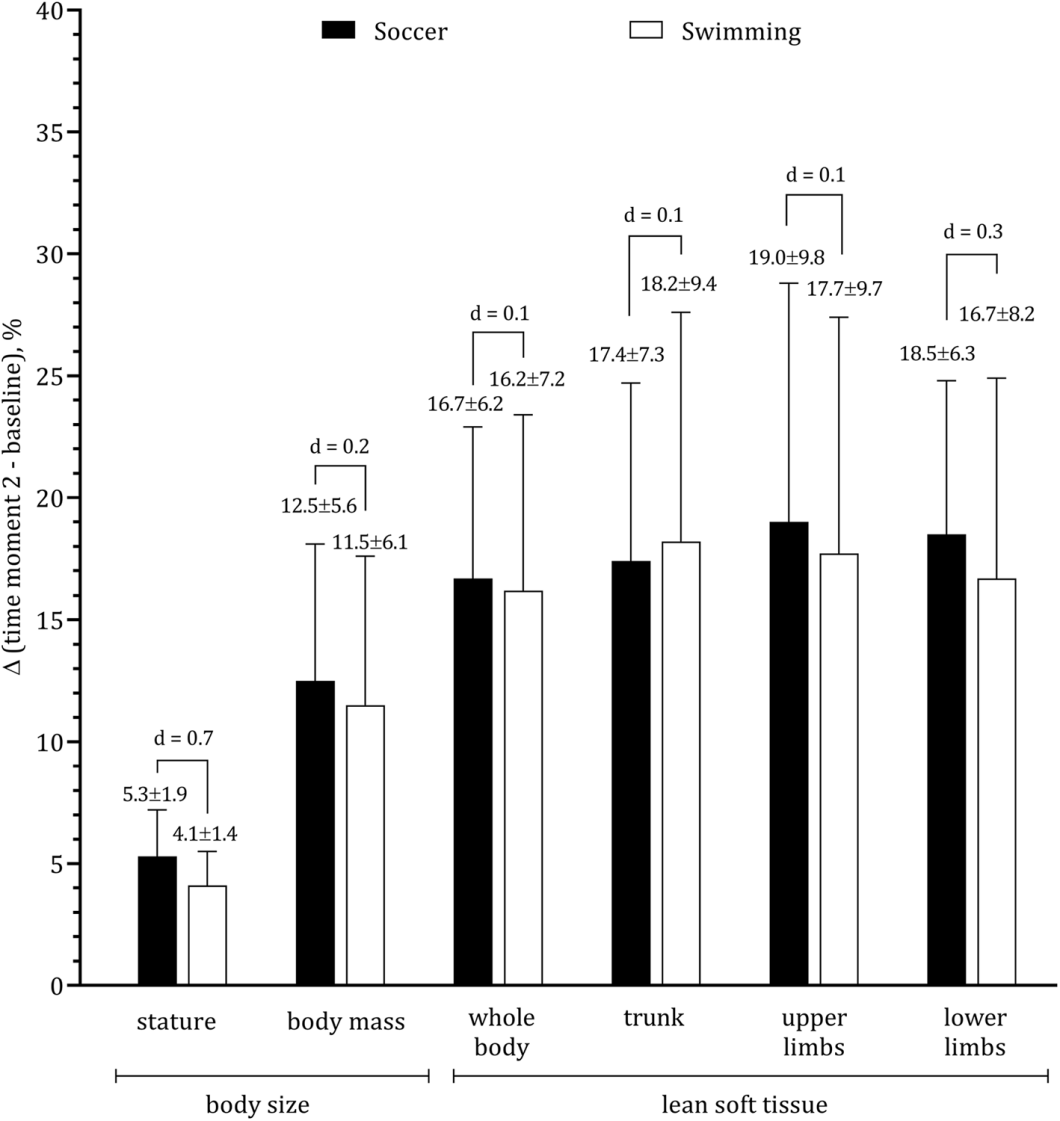
Intra-individual mean changes (% of baseline) for male adolescent soccer players and swimmers on stature, body mass and lean soft tissue

**Fig. 2:**
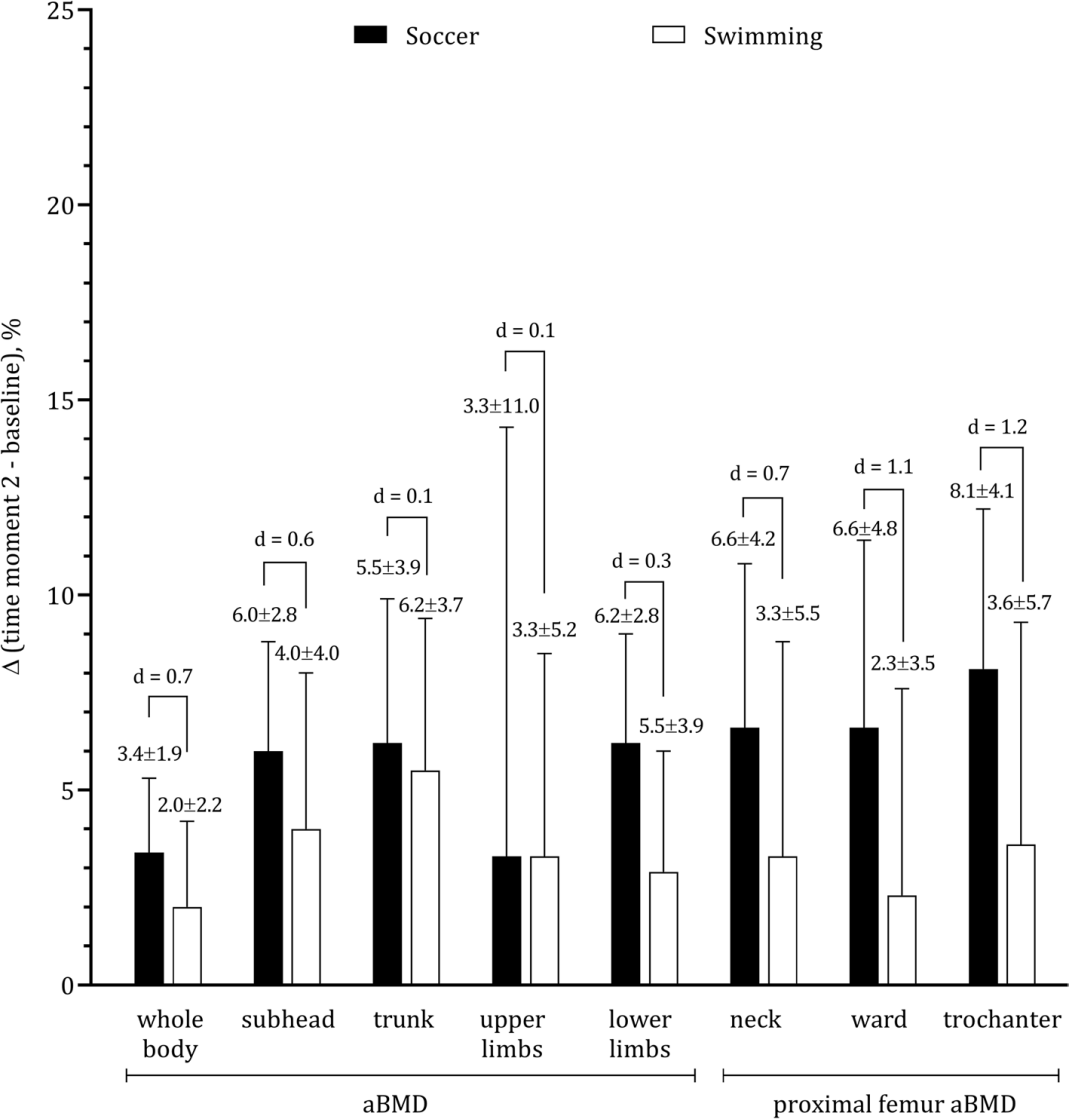
Intra-individual mean changes (% of baseline) for male adolescewnt soccer players and swimmers on areal bone mineral density of whole body, subhead, trunk, upper limbs, lower limbs and proximal femur

Intra-individual mean differences were always significant for body size, body composition and aBMD except for the upper limbs as summarized in Table 2 that also included significant interaction terms for all aBMD sites with the exception of upper limbs. Figure 1 illustrates the intra-individual changes (time moment 2 – baseline) expressed as percentage of baseline values, separately for each sport. The effect size between groups for LST was small for the lower limbs (d = 0.25). When the groups were compared on annual gains in LST on total body, trunk and upper limbs, the differences were negligible. Finally, Fig. 2 evidences a large effect size between soccer players and swimmers (d = 1.2) when the groups were compared in aBMD at the trochanter.

**Table 2:** Results of Repeated measures ANOVA to examine the effects of sports (soccer *vs.* swimming), 12-month follow-up (time-moment 1 *vs.* time-moment 2) and interaction factor (sport ^d^ 12-month) among male adolescent athletes (soccer players, *n* = 20; swimmers, *n* = 20)

Dependent variable	Effects								
	Between sports			12-month			Interaction (sport ^d^ 12-month)		
	F	p	η^2^	F	p	η^2^	F	p	η^2^
	Factorial ANOVA								
Stature, cm	3.472	0.070	0.29^b^	319.355	< 0.001	0.95^f^	4.211	0.047	0.32 ^c^
Body mass, kg	0.989	0.326	0.16^b^	170.417	< 0.001	0.90^f^	0.193	0.663	0.07
Fat tissue, %	3.733	0.061	0.30^c^	9.281	0.004	0.44^c^	1.067	0.192	0.21^b^
LST: total body, kg	7.850	0.008	0.41^c^	223.043	< 0.001	0.92^f^	0.393	0.535	0.10^b^
LST: trunk, kg	8.186	0.007	0.42^c^	177.569	< 0.001	0.91^f^	1.011	0.391	0.16^b^
LST: upper limbs, kg	11.598	0.002	0.48^c^	130.695	< 0.001	0.88^e^	0.472	0.496	0.11^b^
LST: lower limbs, kg	4.371	0.043	0.32^c^	207.302	< 0.001	0.92^f^	0.001	0.989	0.01
aBMD: total body, g.cm^−2^	5.545	0.024	0.36^c^	66.908	< 0.001	0.80^e^	4.847	0.034	0.34^c^
^a^	11.088	0.002	0.48^c^	2.087	0.157	0.23^b^	7.627	0.009	0.41^c^
aBMD: subhead, g.cm^−2^	5.254	0.028	0.35^c^	90.383	< 0.001	0.84^e^	5.195	0.028	0.35^c^
^a^	13.223	0.001	0.51^d^	0.564	0.457	0.12^b^	6.521	0.015	0.39^c^
aBMD: trunk, g.cm^−2^	0.248	0.621	0.08	92.522	< 0.001	0.84^e^	0.459	0.502	0.11^b^
aBMD: upper limbs, g.cm^−2^	0.569	0.455	0.12^b^	1.725	0.197	0.21^b^	0.111	0.741	0.05
aBMD: lower limbs, g.cm^−2^	9.146	0.004	0.44^c^	97.238	< 0.001	0.85^e^	14.294	0.001	0.52^d^
aBMD: PF-neck, g.cm^−2^	5.052	0.030	0.34^c^	40.643	< 0.001	0.72^e^	6.015	0.019	0.37^c^
aBMD: PF-ward, g.cm^−2^	8.129	0.007	0.42^c^	28.575	< 0.001	0.66^d^	9.401	0.004	0.45^c^
aBMD: PF-trochanter, g.cm^−2^	7.718	0.008	0.41^c^	56.514	< 0.001	0.77^e^	11.106	0.002	0.48^c^

*LST* (Lean soft tissue), *aBMD* (Areal bone mineral density), *PF* (Proximal femur), *F* (*F*-value), *p* (Significance value)

^a^(Repeated measures ANCOVA controlling for stature at baseline, centered stature = 152.3 cm)

Magnitude effect: ^b^(small), ^c^(moderate), ^d^(large), ^e^(very large), ^f^(nearly perfect)

Finally, Table 3 reports descriptive statistics for BMI and indicators obtained from FFQ, at baseline, separately for swimmers and soccer players. The two groups did not differ.

**Table 3:** Mean (95% confidence limits of the mean) by sports (soccer *vs.* swimming) and comparisons between groups on training experience, body mass index and indicators obtained from food frequency questionnaire

Dependent variable	units	Soccer		Swimming		t-test	
		Mean	95% CL	Mean	95% CL	t-value	p
Training experience	years	5.2	(4.4; 6.0)	4.4	(3.6; 5.2)	1.411	0.166
Body mass index	kg.m^−2^	18.43	(17.30; 19.57)	18.35	(17.17; 19.52)	0.109	0.914
Calories	kcal	3282	(2298; 4265)	2628	(1892; 3364)	1.113	0.273
Proteins	%	23.7	(20.6; 26.8)	21.6	(19.5; 23.7)	1.169	0.250
Carbohydrates	%	60.6	(55.5; 65.7)	59.6	(56.3; 62.8)	0.364	0.718
Fat	%	15.7	(13.4; 18.0)	18.8	(16.1; 21.6)	-1.821	0.076
Cholesterol	mg	488	(317; 660)	357	(279; 436)	1.456	0.154
Fibres	g	36.9	(17.1; 56.7)	30.6	(21.9; 39.2)	0.615	0.545
Calcium	mg	1822	(1241; 2403)	1146	(701; 1590)	1.935	0.060

*95% CL* (95% confidence limits)

## Discussion

The current sample of male adolescent athletes aged 11.9–13.4 years at baseline was characterized by negative values in the maturity offset. The previous confirmed all participants prior to estimated age at PHV that is consensually considered a crucial mark for bone accrual. Mean values of soccer players on stature-for-age and weight-for-age fluctuated between 25 and 50^th^ percentiles of US reference [27]. Although soccer players were, in general, 4.5 cm shorter and 2.3 kg lighter compared to swimmers, mean differences were not significant for body size descriptors. The mean stature of swimmers plotted above 50^th^ percentile and mean body mass was at percentile 50^th^. The current study also assessed body composition given by predicted fat mass derived from DXA technology. The current sample (16.1% of fat mass) approached a previous study [28] of 81 Portuguese male adolescent soccer players aged 14.61 years (17.3%).

During years of maximal height velocity, fat mass tends to attain a plateau in boys [12]. The average aBMD value in total sample was 0.975 g,cm^−2^ at the TM1 (age: 12.6 ± 0.4 years) which is less than 1.078 g^.^cm^−2^ reported for 28 Portuguese school boys aged 15.9 ± 2.8 years [29]. In the current study, adolescents grouped by sports (soccer *versus* swimming) were assessed twice: in the TM1 and after 12 months. The groups differed in terms of training parameters with substantial more minutes and training sessions completed by swimmers. Not surprisingly, swimmers gained + 4.3 kg of LST with significant differences at all segments (upper limbs, trunk and lower limbs). Regardless of changes in body mass and LST, soccer players were characterized by significant higher aBMD values in the lower limbs. Of interest, aBMD mean values of the two groups did not differ at upper limbs nor trunk. Regarding proximal femur scan, aBMD was consistently higher among soccer players compared to swimmers.

As bones increase in length, they also increase in width, so bone formation and resorption were coordinated to preserve structural strength [30]. Peak bone mass refers to the amount of bone acquired at the end of skeletal development and is viewed as an important determinant of lifespan skeletal health. Inter-individual variance in bone mass, as other tissue outcomes, needs to be interpreted in relation to age and biological maturation. In fact, circumpubertal years (-2 to + 2 years of PHV) are considered the most decisive period for bone mass accumulation, in which approximately 33% and 39% of all BMC observed at adult state is acquired in the femur neck and whole body of boys, respectively [3]. The literature refers the timing of puberty as inversely related to peak bone mass with late-maturing individuals characterized by lower levels of bone mass in young adulthood [31]. In the Saskatchewan Pediatric Bone Mineral Study, BMC was annually measured on six occasions to determine BMC velocity using a cubic spline fit in addition to peak accretion rates [32]. The resulting peak bone mineral accrual rate was, on average, 407 g.year^−1^ for boys which corresponded to a peak calcium accretion rate of 359 mg.day^−1^ (assuming 32.2% as the fraction of calcium in bone mineral). The study of Canadian adolescents estimated 14.0 years as the mean age of peak calcium accretion. Even among children of similar age, those who are tall for age have greater BMC and aBMD than those who are average or short for age [31]. The groups of adolescent boys participating in soccer and swimming did not significantly differ for stature nor body mass and, consequently, increment rates over a 12-month period is mainly explained by type and frequency of participation in sports.

The onset and progression through puberty growth spurt is accompanied by alterations in body composition, particularly an increment in fat-free mass among boys, contrasting to an increase in fat mass that is commonly reported for girls [12]. The variance in body mass on bone accretion if of particular interest because of the beneficial effects of weight-loading on bone accretion. Additionally, muscle contractions generate forces which stimulate bones to adapt their shape and density [33, 34]. Prospective studies have shown that lean body mass accretion was the primary determinant of total body peak BMC accretion, explaining 50% of the variability in peak BMC accretion [30]. Actually, among healthy school boys aged 10–17 years [35], the contribution of lean tissue to BMC ranged 5.7–12.3% depending on the skeletal site and it was also concluded that lean tissue was strongly associated to BMC of the femoral neck, whereas fat mass was particularly associated to BMC of the whole body and lumbar spine. Peak gain in cross-sectional muscle area is an indicator of muscle mass which tend to occur one year after the peak gain in tibia length and earlier than cortical cross-sectional area, total BMC and cortical volumetric BMD assessed by quantitative computed tomography [36]. Thus, quantitative and qualitative changes in bone tissue seemed establish a dynamic interrelationship to changes in body mass and its components (fat and muscle tissues). In the present study, swimmers and soccer players did not differ neither in body mass as previously mentioned nor in fat tissue in contrast to LST that consistently presented higher mean values among swimmers. Thus, sport-associated variation in aBMD emerged as a consequence of sport participation.

Regarding the potential effect of participation in sports on bone health, the literature has been primarily focused on comparisons of aBMD values between athletes and the general population [37]. In the preceding mentioned study, adult rugby players showed greater body mass, and greater total lean and fat masses than controls; in addition, the sample of 20 rugby players aged 23.6 years displayed consistently higher aBMD man values than counterparts: lower limbs (16%), upper limps (21%) and pelvis (17%). In the current study of adolescent participants, mean differences on aBMD at various sites was essentially noted in the lower limbs. In contrast to rugby, physical demands and mechanical impacts in soccer are essentially concentrated in the lower extremities. More recently, another study [17] examined the differences on bone density, geometry, and strength between soccer and non-impact sport (swimming and cycling) among male adolescents. The cross-sectional data of the previously cited study presented soccer players as having higher aBMD in subhead, hip and legs compared to swimmers (differences ranged 6.9–13.9%) and cycling adolescent athletes (5.3–12.7%). Another recent study [38] confirmed lower values of aBMD at lower limbs in male swimmers compared to counterparts participating in karate, judo, basketball and soccer. Finally, adolescent swimmers aged 13.8 years were compared to controls, tennis and soccer players of similar age [39] and although they trained significantly more hours per week, as in the sample of the current study, the differences between swimmers and other athletes were more pronounced in proximal femur.

The current study assessed the non-dominant side. This might be a limitation of the current study. An alternative is given by performing dual hip scans to obtain mean of right and left aBMC [40]. To explore whether there is difference in aBMD at the hip between dominant and non-dominant sides in young adult athletes participating in low- and high-impact sports, measurements of both hips were recorded on 194 athletes of both sexes using dual-energy X-ray absorptiometry [41]. Of relevance, the previous study concluded that aBMD mean values in the dominant hip was higher compared to non-dominant among participants in low impact sports, but not among athletes participating in high impact sports. Meantime, the proximal femur region is commonly assessed by DXA in adults and is considered more challenging to evaluate young people [24]. Although the preceding, data from the Bone Mineral Density in Childhood Study (BMDCS) age-related precision of the total hip and femoral neck as comparable to the spine and total body less head [42].

The present study classified all participants as PHV according to a non-invasive protocol [20]. The algorithm used to predict maturity offset values was derived from Canadian and Belgian longitudinal studies and its applicability has been recently discussed [43–45]. Indeed, the equation to predict age at PHV seemed dependent from CA and body size and inter-individual variance in predicted age at PHV was narrower compared to observed age at PHV. Future research should include a control group to distinguish the impact of participation in sports on bone health. Although limited sample size and absence of a control group, the current 12-month follow-up research consistently confirmed the osteogenic effect of participation in sport as previously suggested in cross-sectional studies [6, 46]. Additional research is needed including measurements of habitual physical activity among non-participants and participants in sports.

## Conclusion

During years of maximal growth, participation in sports is associated to gains in LST and seemed to prevent increments in fat mass. Bone accrual were also notorious in aBMD both in swimmers and soccer players with the magnitude of intra-individual gains more pronounced among soccer participants, particularly in the lower limbs and at proximal femur sites. The present study confirmed pubertal growth spurt as a critical period for aBMD outcomes besides the type of sports. Of particular relevance, participation in sports is often viewed as an important form of physical activity and, additionally, add beneficial adaptations on bone health indicators, particularly in sports characterized by mechanical loading.

## Supplementary Information

**Additional file 1.**

## Data Availability

All data generated or analysed during this study are included in this published article [and its supplementary information files].

## Abbreviations

BMC: Bone mineral content
aBMD: Areal bone mineral density
DXA: Dual energy x-ray absorptiometry
TM1: Baseline
TM2: 12-Month follow up
CA: Chronological age
PHV: Peak height velocity
FFQ: Food frequency questionnaire
ANOVA: Analysis of variance; LST: lean soft tissue
